# Effects of dipolar interactions on the sensitivity of nonlinear spinor-BEC interterometry

**DOI:** 10.1038/s41598-018-21566-9

**Published:** 2018-02-19

**Authors:** Qing-Shou Tan, Qiong-Tao Xie, Le-Man Kuang

**Affiliations:** 1 College of Physics and Electronic Engineering, Hainan Normal University, Haikou, 571158 China; 2 Key Laboratory of Low-Dimensional Quantum Structures and Quantum Control of Ministry of Education, Hunan Normal University, Changsha, 410081 China; 3 Department of Physics, Hunan Normal University, Changsha, 410081 China

## Abstract

We consider the effects of dipole-dipole interactions on a nonlinear interferometer with spin-1 Bose-Einstein condensates. Compared with the traditional atomic SU(1,1) interferometer, the shot-noise phase sensitivity can be beaten with respect to the input total average number of particles; and the improved sensitivity depends on the effective strength of the dipolar interaction via modifying the trapping geometry. It indicates that the best performance of the interferometer is achieved with highly oblate trap potential. The Bayesian phase estimation strategy is explored to extract the phase information. We show that the Cramér-Rao phase uncertainly bound can saturate, when the ideal dis-entangle scheme is applied. The phase average of the phase sensitivity is also discussed.

## Introduction

Interferometers as the extremely useful and flexible precise measuring tool, play a key role in the field of quantum metrology^1–21^. Recently, there are mainly two classes of interferometers^2,3^: passive [e.g., Mach-Zehnder interferometer (MZI)] and active [e.g., SU(1,1)] interferometers. The ultimate goal of both these setups is to beyond the shot-noise limit (SNL) for phase estimation. It is well known that, for the MZI to beat SNL, i.e., $\mathrm{1/}\sqrt{N}$ with *N* being the total particles number, nonclassicality of the input states are necessary. While for SU(1,1) interferometer the situation is different, because it applies the nonlinear optical-parametric amplifier (OPA), which mixes the optical beams and then converts the classical input states into photon pairs^2–4^. A potential advantage of the SU(1,1) interferometer is that even for classical sources of the input states the SNL can also be surpassed.

Spinor Bose-Einstein condensates due to their unique coherence properties and the controlled nonlinearity are viewed as the ideal sources for an atomic interferometer. The coherent spin-mixing dynamics (SMD) in the spin-1 BECs can generate entangled states^22–26^ by converting two atoms in the *m*_*f*_ = 0 state into one atom in the *m*_*f*_ = 1 state and the other in the *m*_*f*_ = −1 state, which is the atomic analogue of OPA. Experimentally, ref.^27^ has used the spin-changing collisions in a spinor BEC as the nonlinear mechanism to realize a atomic SU(1,1) interferometer. In this scheme the interferometer operations belong to the SU(1,1) group and the phase sensitivity can be obtained analytically by using mean-field approximation, but the number of particles used for phase estimation inside the interferometer is very small. To obtain a relatively large number of particles for probe states, in ref.^28^, the authors considered a full quantum analysis and found that the sub-shot-noise(SSN) phase sensitivity can be obtained with respect to the total particles inside the interferometer.

Up to now, studies of nonlinear atomic interferometer with spinor BECs have focused mainly on *s*-wave contact interaction^27–29^. According to the recent experimental and theoretical observation in ^23^Na and ^87^Rb atoms, the magnetic dipole-dipole interactions (MDDIs) are indeed not negligible for these spinor condensates^26,30–39^. For example, in ^87^Rb atoms, the magnitude of the dipolar energy can be as large as 10% of the spin-exchange energy^35,36^. In particular, the long-range and anisotropic nature of the dipolar interaction may further enhance its effects^36,38,40^. Thus, the effects of the MDDI should be considered in a reliable SSN sensitivity interferometer based on spinor BECs.

In this paper, we study the effects of MDDI on the phase sensitivity in a spin-mixing interferometer based on ^87^Rb condensates. In the quantum metrology field, the quantum Fisher information (QFI)^41–43^ has been widely used to characterize the phase sensitivity. In this work, we will also describe the phase sensitivity for our spin-mixing interferometer with the QFI. By calculating the QFI, we find that, the QFI depends on both the evolution time of SMD and the trapping geometry. Our results indicate that, for certain evolution time the enhancement SSN sensitivities can be reached with respect to the total input number of particles *N* by using the highly oblate trap potential. Finally, we also explore the Bayesian phase estimation strategy to extract the optimal phase information and phase average sensitivity with different dis-entangle methods.

## Results

### Model and Hamiltonian

Similar to the optical SU(1,1) interferometer, the spin-mixing interferometer can also divide into three steps: (I) entangled states preparation with spin-exchange collisions, (II) phase encoding, and (III) dis-entangling and measurement.

**Figure 1:**
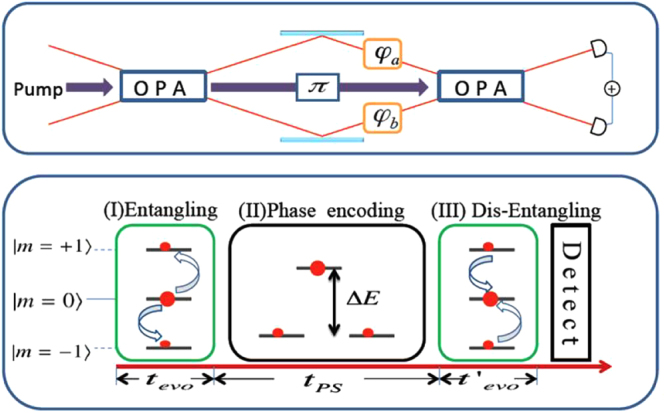
Schematic representation of an optical SU(1,1) interferometer and its correspondent realization in atomic spin-mixing interferometer. For atomic interferometer the unknown phase *θ* = 2Δ*Et*_PS_.

To realize the atom interferometer scheme as shown in Fig. 1, we consider *N* spin-1 Rb atoms confined in a three-dimensional potential with ferromagnetic spin-exchange collisional interaction. Assuming all spin components share a common spatial mode *ϕ*(*r*), under the single-mode approximation, the Bose condensate can be described by following Hamiltonian

1 $$\begin{array}{rcl}H/|c| & = & -{\hat{S}}^{2}+{d}_{s}\mathrm{(3}{\hat{S}}_{z}^{2}-{\hat{S}}^{2}+{\hat{N}}_{0})\\  &  & -3{d}_{n}({\hat{S}}_{x}^{2}-{\hat{S}}_{y}^{2}-{\hat{a}}_{-1}^{\dagger }{\hat{a}}_{1}-{\hat{a}}_{1}^{\dagger }{\hat{a}}_{-1}).\end{array}$$

The first term originates from the *s*-wave contact interaction, which contains the SMD and has been considered for atom SU(1,1) interferometers^27,28^. The last two terms are induced by the dipolar interaction. Notice, here we have used the absolute value of the spin exchange strength $|c|=|({c}_{2}\mathrm{/2)}\,\int \,dr|\varphi (r{)|}^{4}$ as energy unit (the corresponding unit for time is *ħ*/|*c*|), where *c*_2_ = 4*πħ*^2^(*a*_2_ − *a*_0_)/(3 *M*) with *M* being the mass of the atom and *a*_0,2_ the *s*-wave scattering length for two spin-1 atoms in the symmetric channel of the total spin 0 and 2, respectively. For a Gaussian mode function with characteristic lengths *q*_*x*,*y*,*z*_ in *x*, *y*, *z* directions, the rescaled dipolar interaction strengths can be read as^36^

2 $$\begin{array}{rcl}{d}_{s}({\kappa }_{x},{\kappa }_{y}) & = & \frac{4\pi {c}_{d}}{\mathrm{3|}{c}_{2}|}{\kappa }_{x}{\kappa }_{y}\,{\int }_{0}^{\infty }\,t{e}^{-({\kappa }_{x}^{2}+{\kappa }_{y}^{2}){t}^{2}/2}\\  &  & \times {I}_{0}(\frac{1}{2}({\kappa }_{x}^{2}+{\kappa }_{y}^{2}){t}^{2})\,[2-3\sqrt{\pi }t{e}^{{t}^{2}}\,{\rm{erfc}}(t)]dt,\end{array}$$

3 $$\begin{array}{rcl}{d}_{n}({\kappa }_{x},{\kappa }_{y}) & = & \frac{4{\pi }^{\mathrm{3/2}}{c}_{d}}{\mathrm{3|}{c}_{2}|}{\kappa }_{x}{\kappa }_{y}\,{\int }_{0}^{\infty }\,{t}^{2}{e}^{-({\kappa }_{x}^{2}+{\kappa }_{y}^{2}){t}^{2}/2}\\  &  & \times {I}_{1}(\frac{1}{2}({\kappa }_{x}^{2}+{\kappa }_{y}^{2}){t}^{2}){e}^{{t}^{2}}\,{\rm{erfc}}(t)dt,\end{array}$$

where (*κ*_*x*_, *κ*_*y*_) ≡ (*q*_*x*_/*q*_*z*_, *q*_*y*_/*q*_*z*_) characterizes the shape of the condensate, ${c}_{d}={\mu }_{0}{\mu }_{B}^{2}{g}_{F}^{2}\mathrm{/(4}\pi )$ is the strength of the MDDI with *μ*_*B*_ the Bohr magneton, and *g*_*F*_ the Landé *g*-factor for ^87^Rb atoms, we have *c*_*d*_/|*c*_2_| ≈ 0.1. In the above equations, *I*_0,1_(*x*) is the modified Bessel functions of the first kind, and erfc(*x*) is the complementary error function. The value of *d*_*s*,*n*_ can be positive, 0, or negative, depending on the values of *κ*_*x*,*y*_. In particular, *d*_*s*,*n*_ = 0 (*d*_*n*_ = 0) if *κ*_*x*_ = *κ*_*y*_ = 1 (*κ*_*x*_ = *κ*_*y*_).

In Eq. (1), the many-body angular momentum operators are given by

4 $$\begin{array}{rcl}{\hat{S}}^{2} & = & {({\hat{N}}_{1}-{\hat{N}}_{-1})}^{2}+\mathrm{(2}{\hat{N}}_{0}-\mathrm{1)}({\hat{N}}_{1}+{\hat{N}}_{-1})\\  &  & +\mathrm{2(}{\hat{a}}_{1}^{\dagger }{\hat{a}}_{-1}^{\dagger }{\hat{a}}_{0}^{2}+h.c),\\ {\hat{S}}_{x} & = & \frac{\sqrt{2}}{2}[{\hat{a}}_{0}^{\dagger }({\hat{a}}_{-1}+{\hat{a}}_{1})+h.c],\\ {\hat{S}}_{y} & = & \frac{\sqrt{2}}{2i}[{\hat{a}}_{0}^{\dagger }({\hat{a}}_{-1}-{\hat{a}}_{1})-h.c],\end{array}$$

with ${\hat{a}}_{\alpha =0,\pm 1}$ being the annihilation operator of the *α*-th spin state, and ${\hat{N}}_{\alpha }$ is the number operator of the *α* spin component. The term $({\hat{a}}_{1}^{\dagger }{\hat{a}}_{-1}^{\dagger }{\hat{a}}_{0}^{2}+h.c)$ include in ${\hat{S}}^{2}$ is identical to the OPA in nonlinear optics, which is the main factors influencing the spin-mix process.

As shown in step (I), we start with a source of *N* = *N*_0_ particles in the *m*_*f*_ = 0 pure state, namely |Ψ(0)〉 = |0, *N*, 0〉 with Fock basis |*N*_1_, *N*_0_, *N*_−1_〉. Governing by Hamiltonian (1), at time *t*_evo_ we can obtain an entangled state $|{\rm{\Psi }}({t}_{{\rm{evo}}})\rangle ={\sum }_{m,k}\,{\bar{g}}_{mk}({t}_{{\rm{evo}}})|k,N-2k+m,k-m\rangle$, where ${\bar{g}}_{mk}({t}_{{\rm{evo}}})$ can be obtained numerically (see Methods).

And then the phase information is encoded into this probe state [step (II)]. To eliminate the effects of atomic nonlinear interaction on the phase accumulation and measurement, we need the accurately controllable spin-changing collisions. In the present of strong enough external magnetic field, the spin-mixing process would be stopped due to the so-called quadratic Zeeman effect. It shifts the the levels of the *f* = 1 down, and induces the energy difference between the *m*_*f*_ = 0 and *m*_*f*_ = ±1 modes via the supplemented Hamiltonian ${H}_{{B}^{2}}=q({\hat{N}}_{1}+{\hat{N}}_{-1})$^27,28^. In ref.^27^, the authors obtained energy difference *q* = (2*π*)72 Hz when *B* = 0.9 G. Note that the linear Zeeman effect does not affect the spin-changing collisions since the energy gained by one particle has to be spent by the other. Although spin-changing might be turned on by quenching the magnetic field down to zero, ramping up and down magnetic fields lacks the necessary control and speed. Experimentally, instead we can use microwave dressing to compensate the magnetic field during *t*_evo_, by applying a far-off-resonate detuned *π*-polarized microwave field to coupe |1, 0〉 to |2, 0〉 with the Rabi frequency Ω and the detuning Δ^27^. Microwave dressing supplements the Hamiltonian with ${H}_{{\rm{\Omega }}}=\frac{{{\rm{\Omega }}}^{2}}{4{\rm{\Delta }}}({\hat{N}}_{1}+{\hat{N}}_{-1})$, which can shift up and down the energy level by either red or blue detuning. Making ${{\rm{\Omega }}}^{2}\mathrm{/4}{\rm{\Delta }}=\mathrm{(1}+{d}_{s})\mathrm{(2}{\hat{N}}_{0}-\mathrm{1)}$ during *t*_PS_, the effective interaction of linear phase shift reads:

5 $${H}_{{\rm{PS}}}/|c|=q({\hat{N}}_{1}+{\hat{N}}_{-1})-6{d}_{n}{\hat{N}}_{0}({\hat{a}}_{1}^{\dagger }{\hat{a}}_{-1}+{\hat{a}}_{-1}^{\dagger }{\hat{a}}_{1}),$$

with phase shift *θ* = 2*qt*_PS_.

In step (III), to estimate the phase shift *θ*, we should first dis-entangling the *m*_*f*_ = ±1 modes, and then measure the number of particles in them. An ideal method to dis-entangling is to make *Ht*_evo_ = −*H*′*t*′_evo_. Thanks to both the sign and the strength of the non-linear coupling are experimentally adjustable, which indicates that we maybe make $c{t}_{{\rm{evo}}}\simeq -\tilde{c}{t^{\prime} }_{{\rm{evo}}}$ and ${c}_{d}{t}_{{\rm{evo}}}\simeq -{\tilde{c}}_{d}{t}_{{\rm{evo}}}$ to realize time reversal read-out scheme.

We can see in all the above three steps, the MDDI plays a important role, later we shall study the effects of the MDDI on the precision of phase estimation in the dipolar atomics interferometer.

### Quantum Fisher information in the present of dipolar interaction

Now, we investigate the effect of MDDI on the phase estimation by calculating the QFI. It gives a theoretically achievable limit on the precision of an unknown parameter *θ* by the quantum Cramér-Rao theorem Δ^2^*θ* ≥ Δ^2^*θ*_*QCR*_ = 1/(*mF*_*Q*_), where *m* represents the number of independent measurements. In our interferometer the QIF can be obtained as

6 $$\begin{array}{rcl}{F}_{Q} & = & 4\,{\rm{Var}}({\hat{N}}_{s}\mathrm{/2})\\  & = & \langle {\rm{\Psi }}({t}_{{\rm{evo}}})|{\hat{N}}_{s}^{2}|{\rm{\Psi }}({t}_{{\rm{evo}}})\rangle -{\langle {\rm{\Psi }}({t}_{{\rm{evo}}})|{\hat{N}}_{s}|{\rm{\Psi }}({t}_{{\rm{evo}}})\rangle }^{2}\end{array}$$

with ${\hat{N}}_{s}={\hat{N}}_{1}+{\hat{N}}_{-1}$. For convenience, we define the mean quantum Fisher information ${\bar{F}}_{Q}={F}_{Q}/N$, where $\bar{F}=1$ means the SNL and ${\bar{F}}_{Q} > 1$ means the SSN phase sensitivity.

**Figure 2:**
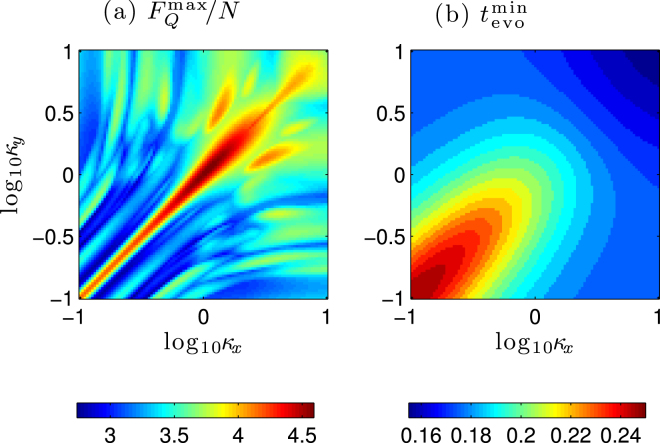
(**a**) The maximal mean QFI and (**b**) the shortest time to obtain SSN for different *κ*_*x*_, *κ*_*y*_ with total atomic number *N* = 20, for ^87^Rb one has *c*_*d*_/|*c*_2_| = 0.1.

In Fig. 2(a), we have plotted the maximal mean QFI ${\bar{F}}_{Q}^{{\rm{\max }}}$ with long enough evolution time for different *κ*_*x*_, *κ*_*y*_. As it is shown, we have ${\bar{F}}_{Q}^{{\rm{\max }}} > 1$, which depends on the trapping geometry (*κ*_*x*_, *κ*_*y*_); and the best QFI is appeared in the regimes of *κ*_*x*_ = *κ*_*y*_, corresponding to the axial symmetry with *d*_*n*_ = 0. It indicates that we can obtain the SSN sensitivity with respects to the total atom number *N*. However, in Fig. 2(a) the maximal QFI is attained for long enough evolution times, and hence the mechanism of decoherence in the condensate cannot be neglected. In fact, to reach the SSN sensitivities long evolution time is not necessary. In Fig. 2(b), we have shown the shortest evolution time to reach the SSN sensitivities as a function of *κ*_*x*_, *κ*_*y*_. It is clearly shown that the MDDI can reduce the evolution time to obtain the SSN.

To avoid the mechanism of decoherence and obtain the relatively large QFI, in the spin-mixing interferometer the maximum SSD evolution time we considered is mainly focus on the scale of $\sim \hslash /(|c|\sqrt{N})$, which is much shorter than the lifetime of a spin-1 BECs. And hence, for sufficiently large *N* and fast phase encoding the nonlinear interferometer, we can safely neglect the decoherence processes of the condensate.

**Figure 3:**
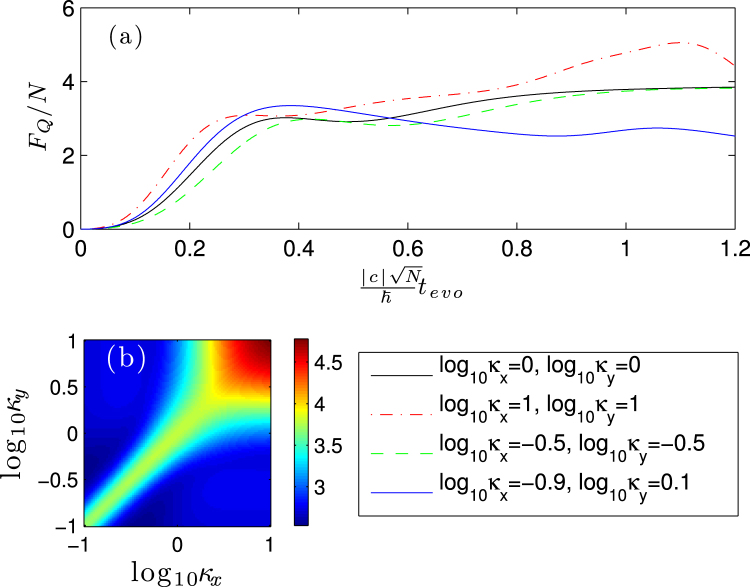
(**a**) The mean QFI as a function of evolution time for different (*κ*_*x*_, *κ*_*y*_). (**b**) The dependence of the mean QFI on the trapping geometry (*k*_*x*_, *k*_*y*_) when ${t}_{{\rm{evo}}}=\hslash /(|c|\sqrt{N})$. The parameters are chosen as *N* = 30 and *c*_*d*_/|*c*_2_| = 0.1.

In Fig. 3(a), we have plotted the mean QFI as a function of evolution time for different *κ*_*x*_, *κ*_*y*_. As it is shown, we can find that for short time scale the QFI increase with time evolution, it means that proper period of evolution time can improve the QFI, and the values can surpass the SNL. Figure 3(a) indicates that due to the MDDI we can obtain better QFI than the case without it (i.e., *κ*_*x*_ = *κ*_*y*_ = 1). In particular, we can obtain the best QFI when ${\mathrm{log}}_{10}\,{\kappa }_{x}={\mathrm{log}}_{10}\,{\kappa }_{y}=1$, i.e., pancake-shaped condensate. In Fig. 3(b) we plot the dependence of the mean QFI on the trapping geometry (*κ*_*x*_, *κ*_*y*_) covering the parameter regime 0.1 ≤ *κ*_*x*,*y*_ ≤ 10 with evolution time $|c|\sqrt{N}\hslash {t}_{{\rm{evo}}}/\hslash =1$. It is shown large QFI can find in the regime *κ*_*x*_ = *κ*_*y*_, corresponding to the axial symmetry with *d*_*n*_ = 0. The diagonal lines in Fig. 3 illustrate the changes of the QFI when the condensate changes from the elongated trap (cigar-shaped) to oblate trap (pancake-shaped). In both Fig. 3(a,b), the best QFIs are found in the region with highly oblate ${\mathrm{log}}_{10}\,{\kappa }_{x}={\mathrm{log}}_{10}\,{\kappa }_{y}=1$. This means that we can obtain the best SSN sensitivities with respect to the total input number of particles *N*, which is larger than the case without the MDDI, by initially setting the shape of the condensate.

The mechanism of the MDDI improves the phase sensitivity in short time scales can be understood from Hamiltonian (1). After rescaling Eq. (1), we have

7 $$ {\mathcal H} ={\hat{S}}^{2}+{A}_{x}{\hat{S}}_{x}^{2}+{A}_{y}{\hat{S}}_{y}^{2}+E({\hat{a}}_{1}^{\dagger }{\hat{a}}_{-1}+{\hat{a}}_{-1}^{\dagger }{\hat{a}}_{1}\mathrm{)}.$$

**Figure 4:**
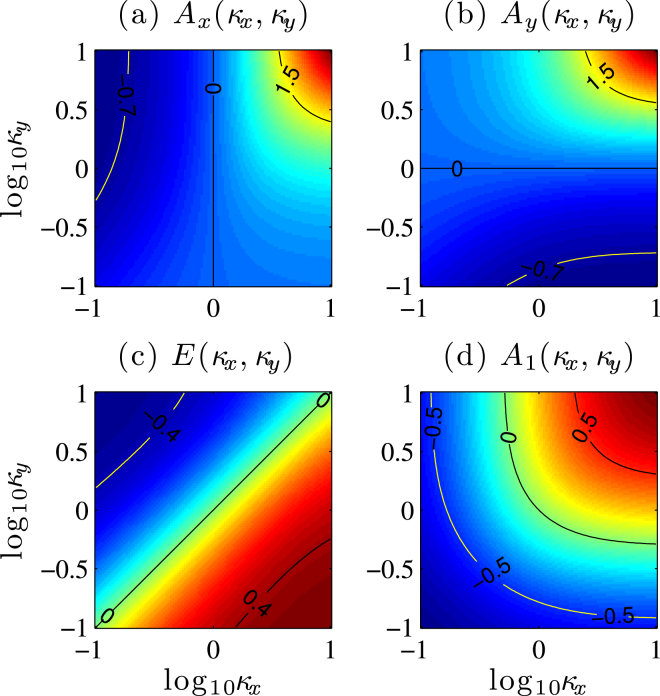
Anisotropic constants *A*_*x*_(*κ*_*x*_, *κ*_*y*_), *A*_*y*_(*κ*_*x*_, *κ*_*y*_), *E*(*κ*_*x*_, *κ*_*y*_) and *A*_1_(*κ*_*x*_, *κ*_*y*_) for ^87^Rb, where *c*_*d*_/|*c*_2_| = 0.1.

The anisotropic constants are given by

8 $${A}_{x}=\frac{\mathrm{3(}{d}_{s}+{d}_{n})}{1-2{d}_{s}},\quad {A}_{y}=\frac{\mathrm{3(}{d}_{s}-{d}_{n})}{1-2{d}_{s}},\quad E=\frac{3{d}_{n}}{1-2{d}_{s}},$$

which depend on the MDDI. The values of *A*_*x*_(*κ*_*x*_, *κ*_*y*_), *A*_*y*_(*κ*_*x*_, *κ*_*y*_) and *E*(*κ*_*x*_, *κ*_*y*_) covering the parameter regime 0.1 ≤ *κ*_*x*,*y*_ ≤ 10 are shown in Fig. 4. According to Fig. 4, we have *A*_*x*_ ≥ 0 (*A*_*y*_ ≥ 0) if *κ*_*x*_ ≥ 1 (*κ*_*y*_ ≥ 1). In particular, if *κ*_*x*_ = *κ*_*y*_ we have *E* = 0 and *A*_*x*_ = *A*_*y*_, then Eq. (1) further reduces to ${\mathcal H} ^{\prime} ={\hat{S}}^{2}-{A}_{1}{\hat{S}}_{z}^{2}$ with ${A}_{1}=\frac{3{d}_{s}}{1+{d}_{s}}$ and ${\hat{S}}_{z}={\hat{a}}_{1}^{\dagger }{\hat{a}}_{1}-{\hat{a}}_{-1}^{\dagger }{\hat{a}}_{-1}$.

**Figure 5:**
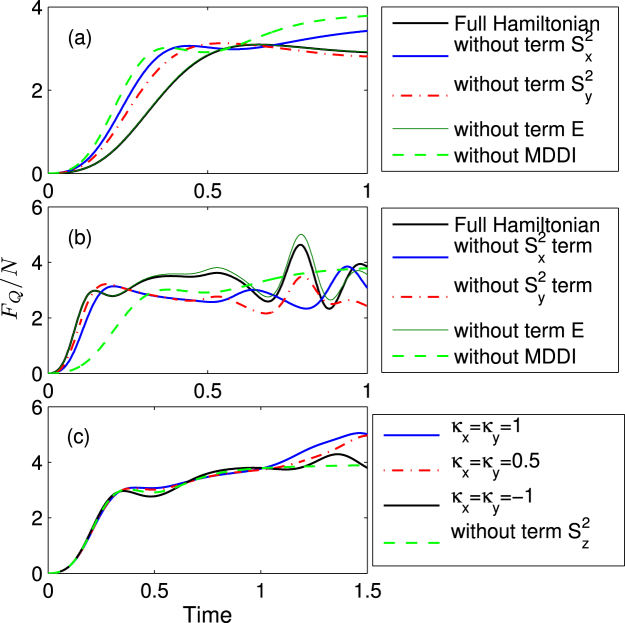
Comparisons of mean QFI with and without the MDDI. (**a**) ${\mathrm{log}}_{10}\,{\kappa }_{x}=-0.8$, ${\mathrm{log}}_{10}\,{\kappa }_{y}=-0.5$ corresponding to *A*_*x*_ = −0.533, *A*_*y*_ = −0.228 and *E* = −0.153. (**b**) ${\mathrm{log}}_{10}\,{\kappa }_{x}=0.9$, ${\mathrm{log}}_{10}\,{\kappa }_{y}=0.5$ corresponding to *A*_*x*_ = −1.64, *A*_*y*_ = 1.26 and *E* = 0.194. Here, *N* = 30.

Now we will investigate the effects of anisotropic constants *A*_*x*_(*κ*_*x*_, *κ*_*y*_), *A*_*y*_(*κ*_*x*_, *κ*_*y*_), *E*(*κ*_*x*_, *κ*_*y*_) and *A*_1_(*κ*_*x*_, *κ*_*y*_) on the QFI with rescaled times, respectively. From Fig. 5, we can find that the values of the mean QFIs almost has no influence on the parameter *E*, but it significantly depends on the values of *A*_*x*,*y*_. That is, the positive values of *A*_*x*,*y*_ can improve the QFI for short time scales, while decrease it for negative values of *A*_*x*,*y*_. Figure 5(c) shows the QFI in an atomic interferometer based on axial-symmetry condensate, from it we can find that the term including ${S}_{z}^{2}$ nearly do not affect the QFI within short time scales, but it can enhance the QFI for relatively long time time scales.

It is well known that the SNL of phase sensitivity can be surpassed using squeezed states. Later, we will investigate the effects of the condensate shape on the phase sensitivity by calculating the spin squeezing in the system considered. Unlike the spin-1/2 systems which can be uniquely specified by different components of the total spin vector **S** ≡ (*S*_*x*_, *S*_*y*_, *S*_*z*_), the state of spin-1 atomic Bose-Einstein condensates is specified in terms of both the spin vector and nematic tensor *Q*_*ij*_ = *S*_*i*_*S*_*j*_ + *S*_*j*_*S*_*i*_ − (4/3)*δ*_*ij*_ which constitutes SU(3) Lie algebra, with *δ*_*ij*_ being the Kronecker delta and ({*i*, *j*} ∈ {*x*, *y*, *z*})^44,45^. For the initial state we considered, we can always numerical check that $\langle {\bf{S}}\rangle \simeq 0$, but the quadrupole elements 〈*Q*_*ii*_〉 ≠ 0. And in both of the subspaces, {*S*_*x*_, *Q*_*yz*_, *Q*_+_} and {*S*_*y*_, *Q*_*xz*_, *Q*_–_} that exhibit squeezing, where *Q*_+_ and *Q*_−_ are defined *Q*_+_ = *Q*_*zz*_ − *Q*_*yy*_ and *Q*_−_ = *Q*_*xx*_ − *Q*_*zz*_, respectively. Then the two different spin-nematic squeezing parameters in an SU(2) subspace are defined by ${\xi }_{x(y)}^{2}={{\rm{\min }}}_{\theta }\,\langle {[{\rm{\Delta }}(\cos \theta {S}_{x(y)}+\sin \theta {Q}_{yz(xz)})]}^{2}\rangle /\langle {Q}_{\pm }\mathrm{/2}\rangle$, with *θ* being the quadrature angle^45^. If ${\xi }_{x(y)}^{2} < 1$ indicates spin-nematic squeezing.

**Figure 6:**
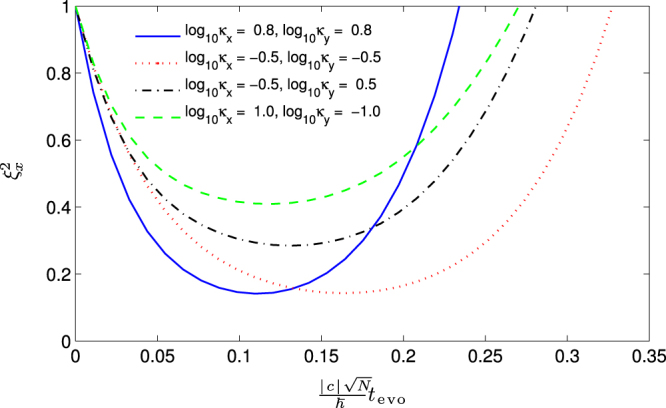
Evolution of spin-nematic squeezing parameter ${\xi }_{x}^{2}$ for different trapping geometry (*κ*_*x*_, *κ*_*y*_) with *N* = 30.

In Fig. 6, we plot the evolution of spin-nematic squeezing parameter ${\xi }_{x}^{2}$ for different trapping geometry. From Fig. 6, we can see that the strong spin-nematic squeezing can be obtained in the regimes of *κ*_*x*_ = *κ*_*y*_, which displays the same changing trend as the QFI. Figure 6 also shows that the highly oblate trap can shorten the optimal squeezing time before it “over squeezing”. In ref.^45^, the authors proposed a scheme to store the best spin-nematic squeezing for quantum metrology by applying periodic microwave pulses.

### Optimal Fisher information in the presence of dipolar interaction

Below, we focus on the atomic interferometer with axial-symmetry condensate, *κ*_*x*_ = *κ*_*y*_. To demonstrate the feasibility of SSN phase sensitivity given by the QFI, we employ a protocol based on a Bayesian analysis of the measurement results with atom-number *N*_±1_ after step (III). Then the classical Fisher information (CFI) is^14^

$${F}_{C}(\theta )=\sum _{{N}_{\pm 1}=0}^{\infty }\,\frac{1}{P({N}_{\pm 1}|\theta )}\,{(\frac{\partial P({N}_{\pm 1}|\theta }{\partial \theta })}^{2},$$

where $P({N}_{\pm 1}|\theta )={|\langle {N}_{\pm 1}|{{\rm{\Psi }}}_{{\rm{out}}}^{(\theta )}\rangle |}^{2}$ is the conditional probability that particle *N*_±1_ is measured for given phase shift *θ*. And $|{{\rm{\Psi }}}_{{\rm{out}}}^{(\theta )}\rangle ={e}^{-iH^{\prime} {t^{\prime} }_{{\rm{evo}}}}{e}^{-i{H}_{{\rm{PS}}}{t}_{{\rm{PS}}}}{e}^{-iH{t}_{{\rm{evo}}}}|{\rm{\Psi }}\mathrm{(0)}\rangle$. Then the saturable lower bound of phase sensitivity is given by the CR bound, ${\rm{\Delta }}{\theta }_{CR}=1/\sqrt{m{F}_{C}(\theta )}$, *m* denotes the number of independent measurements. Unlike the QFI, the CFI depends on the phase *θ*, and by this definition, we have Δ*θ*_*QCR*_ ≤ Δ*θ*_*CR*_.

**Figure 7:**
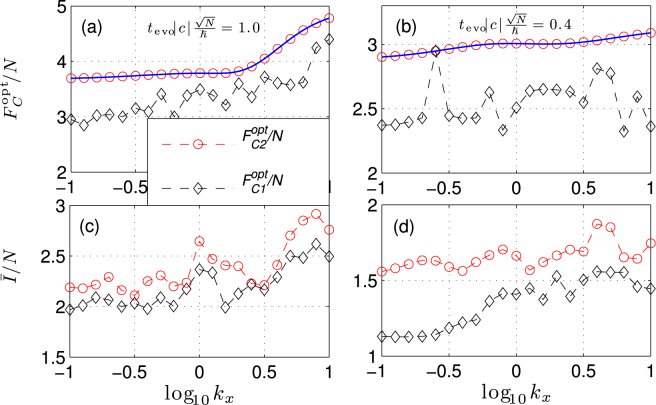
Panels (a,b) Comparison of the optimal CFI with different dis-entangle methods under different evolution time. The blue solid line corresponds to the QFI. Panels (c,d) Comparison of the corresponding phase average of the CFI with different dis-entangle methods. Where ${F}_{C1}^{{\rm{opt}}}$ corresponds to the optimal CFI obtained by the ideal dis-entangle scheme and ${F}_{C2}^{{\rm{opt}}}$ stands for the imperfect dis-entangle. Other parameters are chosen as *N* = 30, *κ*_*y*_ = *κ*_*x*_.

Figure 7 illustrates the optimal CFI ${F}_{C}^{{\rm{opt}}}\equiv {{\rm{\max }}}_{\theta }\,{F}_{C}(\theta )$ as a function of trapping geometry *κ*_*x*,*y*_. Here, we consider *κ*_*x*_ = *κ*_*y*_, then *d*_*n*_ = 0. In Fig. 6, we compare the optimal CFI with two different dis-entangle methods. The first approach to dis-entangling, which considered in ref.^28^, is to apply a *π*/2 phase shift to the *m*_*f*_ = 0 mode, namely ${\hat{a}}_{0}\to i{\hat{a}}_{0}$. After this operation, the many-body angular momentum operators in Eq. (1) become ${S}^{2}\to \tilde{{\hat{S}}^{2}}$ and ${\hat{S}}_{x}^{2}-{\hat{S}}_{y}^{2}\to \tilde{{\hat{S}}_{x}^{2}}-\tilde{{\hat{S}}_{y}^{2}}$ with

9 $$\begin{array}{rcl}\tilde{{\hat{S}}^{2}} & = & {({\hat{N}}_{1}-{\hat{N}}_{-1})}^{2}+\mathrm{(2}{\hat{N}}_{0}-\mathrm{1)}\,({\hat{N}}_{1}+{\hat{N}}_{-1})\\  &  & -\mathrm{2(}{\hat{a}}_{1}^{\dagger }{\hat{a}}_{-1}^{\dagger }{\hat{a}}_{0}^{2}+h.c)\end{array}$$

10 $$\begin{array}{rcl}\tilde{{\hat{S}}_{x}^{2}}-\tilde{{\hat{S}}_{y}^{2}} & = & \{\mathrm{(2}{\hat{N}}_{0}+\mathrm{1)}\,({\hat{a}}_{1}^{\dagger }{\hat{a}}_{-1}+{\hat{a}}_{1}{\hat{a}}_{-1}^{\dagger })\\  &  & -[({\hat{a}}_{1}^{\dagger 2}+{\hat{a}}_{-1}^{\dagger 2}){\hat{a}}_{0}^{2}+h.c.]\}.\end{array}$$

The advantage of this scheme is that it experimentally implement easily. As shown in Fig. 7, we can see that the MDDI can induce better CFI. And we can obtain the SSN phase uncertainties with respect to the total input number of atoms *N*. However, under this scheme, the optical CFI cannot reach the values given by QFI, due to the imperfect dis-entangle. If one want to obtain the optimal phase sensitivity, we can implement the ideal dis-entangle method by changing the sign of *c* and *c*_*d*_, i.e., $c\to -\tilde{c}$ and ${c}_{d}\to -{\tilde{c}}_{d}$. The values of *c* can be controlled via Feshbach resonance, and the value of *c*_*d*_ can be tuned from ${c}_{d}\to {\tilde{c}}_{d}\in [-\mathrm{1/2},1]{c}_{d}$ by using a rotating orienting field to the dipole moments^46^. Note that for perfected dis-entangle scheme, we can also apply the Loschmidt echo protocol to get the optimal phase sensitivity given by the QFI^43^.

To describe well the behaviors of phase estimation for different phase with *θ* ∈ [0, 2*π*], we use the phase average of the FI, which is given by

11 $$\bar{I}=\frac{1}{2\pi }\,{\int }_{0}^{2\pi }\,{F}_{C}(\theta )d\theta .$$

Figure 7(c,d) indicate that for these two dis-entangle methods the phase average of the CFI $\bar{I}$ both can surpass the SQL. From Fig. 7, we can see that in the dipolar atomic spin-mixing interferometer, both the optimal and phase average CFI can reach the SNN limit as long as choose proper evolution time. The phase sensitivities depends on the trapping geometry, and highly oblate trap potential can further improve the average phase estimation precision.

## Discussion

In summary, we have studied a nonlinear interferometer with the dipolar spin-1 Bose-Einstein condensate. By calculating the QFI, we found that the phase sensitivity of the interferometer depends on both the SMD evolution time and the MDDI. It is indicated that proper period of evolution time can improve the QFI, and that the sub-shot-noise phase sensitivity with respect to the total input number of particles *N* can achieve, due to the high transfer rates of particles in the spin-changing process. Moreover, for fixed SSD evolution time, the optimal phase estimation precision is mainly determined by the strength and the sign of the effective dipolar interaction. Our results shown that, the enhancement phase sensitivity can be achieved by tuning the effective MDDI via modifying the trapping geometry. It is indicated that the best performance of the interferometer is achieved with highly oblate trap potential. We also explored the Bayesian phase estimation strategy to extract the phase information. It is shown that the Cramér-Rao phase uncertainly bound can saturate, when the ideal dis-entangle scheme is applied within the time scales that the particle loss effects can be neglected^28^. The phase average uncertainly is discussed, which can also achieve the SSN sensitivity.

Finally, it should be pointed out that the results we have obtained in this paper are based on spin-1 ^87^Rb Bose-Einstein condensate. Indeed, the larger the dipole moment is, the greater the effect is on the nonlinear interferometer. In experiments, dipolar BECs have been realized for atoms with large magnetic dipole moments, such as ^164^Dy with dipole moment 10 *μ*_*B*_, which is much larger than ^87^Rb’s moment equal *μ*_*B*_^47^. Therefore, it will result in the strength of MDDI comparable with the *s*-wave contact interaction in Dy atomic condensate. In ref.^48^, we have investigated the improved spin squeezing induced by MDDI of scalar Dy atomic condensate trapped in a double-well potential, which is useful resource for quantum metrology. In its spinor counterpart, the ground state of Dy atom is ^5^I_8_ with zero nuclear spin, which is spin-8 dipolar condensate. Exploring such complex collisional behavior of Dy atom requires further investigation, but it may be greatly aid attempts in spin-mixing interferometry.

## Methods

### The derivation of Hamiltonian (1)

In the second quantized form, the total Hamiltonian of the system, including *s*-wave collisions and the MDDI, reads as

12 $$H={H}_{0}+{H}_{d},$$

where

13 $$\begin{array}{rcl}{H}_{0} & = & \int \,d{\bf{r}}{\psi }_{\alpha }^{\dagger }({\bf{r}})[-\frac{{\hslash }^{2}{\nabla }^{2}}{2M}+{V}_{ext}({\bf{r}}){\delta }_{\alpha \beta }]{\psi }_{\beta }({\bf{r}})\\  &  & +\frac{{c}_{0}}{2}\int \,d{\bf{r}}{\psi }_{\alpha }^{\dagger }({\bf{r}}){\psi }_{\beta }^{\dagger }({\bf{r}}){\psi }_{\alpha }({\bf{r}}){\psi }_{\beta }({\bf{r}})\\  &  & +\frac{{c}_{2}}{2}\int \,d{\bf{r}}{\psi }_{\alpha }^{\dagger }({\bf{r}}){\psi }_{\alpha ^{\prime} }^{\dagger }({\bf{r}}){{\bf{F}}}_{\alpha \beta }\cdot {{\bf{F}}}_{\alpha ^{\prime} \beta ^{\prime} }{\psi }_{\beta }({\bf{r}}){\psi }_{\beta ^{\prime} }({\bf{r}})\end{array}$$

is the Hamiltonian excluding MDDI. And the dipole-dipole interaction term is

14 $$\begin{array}{rcl}{H}_{d} & = & \frac{{c}_{d}}{2}\,\int \,\frac{d{\bf{r}}d{\bf{r}}^{\prime} }{{|{\bf{r}}-{\bf{r}}^{\prime} |}^{3}}[{\psi }_{\alpha }^{\dagger }({\bf{r}}){\psi }_{\alpha ^{\prime} }^{\dagger }({\bf{r}}^{\prime} ){{\bf{F}}}_{\alpha \beta }\cdot {{\bf{F}}}_{\alpha ^{\prime} \beta ^{\prime} }{\psi }_{\beta }({\bf{r}}){\psi }_{\beta ^{\prime} }({\bf{r}}^{\prime} )\\  &  & -3{\psi }_{\alpha }^{\dagger }({\bf{r}}){\psi }_{\alpha ^{\prime} }^{\dagger }({\bf{r}}^{\prime} )\,({{\bf{F}}}_{\alpha \beta }\cdot {\bf{e}})\,({{\bf{F}}}_{\alpha ^{\prime} \beta ^{\prime} }\cdot {\bf{e}}){\psi }_{\beta }({\bf{r}}){\psi }_{\beta ^{\prime} }({\bf{r}}^{\prime} )],\end{array}$$

with **e** = (**r** − **r**′)/|**r** − **r**′| an unit vector.

Substituting *ψ*_*α*_(**r**) = *a*_*α*_*ϕ*(**r**) into the Hamiltonian, we get

15 $${H}_{0}=c({{\bf{S}}}^{2}-2N),$$

where $c=({c}_{2}\mathrm{/2)}\,\int \,dr|\varphi (r{)|}^{4}$ is the spin-exchange interaction strength, and ${\bf{S}}={a}_{\alpha }^{\dagger }{{\bf{F}}}_{\alpha \beta }{a}_{\beta }$ is the total many-body angular momentum operator. And the dipole-dipole interaction can reduce to

16 $$\begin{array}{rcl}{H}_{d} & = & \frac{{c}_{d}}{2}\,\int \,\frac{d{\bf{r}}d{\bf{r}}^{\prime} {|\varphi ({\bf{r}})|}^{2}{|\varphi ({\bf{r}}^{\prime} )|}^{2}}{{|{\bf{r}}-{\bf{r}}^{\prime} |}^{3}}[{{\bf{S}}}^{2}-\mathrm{3(}{\bf{S}}\cdot {\bf{e}}{)}^{2}\,-\,\mathrm{(2}N-3{a}_{\alpha }^{\dagger }{{\bf{F}}}_{\alpha \beta }\cdot {\bf{e}}{{\bf{F}}}_{\alpha ^{\prime} \beta ^{\prime} }\cdot {\bf{e}}{a}_{\beta })]\\  & = & \frac{{c}_{d}}{2}\,\int \,\frac{d{\bf{r}}d{\bf{r}}^{\prime} {|\varphi ({\bf{r}})|}^{2}{|\varphi ({\bf{r}}^{\prime} )|}^{2}}{{|{\bf{r}}-{\bf{r}}^{\prime} |}^{3}}[({S}_{z}^{2}+{a}_{0}^{\dagger }{a}_{0}-\frac{1}{4}({S}_{+}{S}_{-}+{S}_{-}{S}_{+})\\  &  & -\frac{1}{2}({a}_{1}^{\dagger }{a}_{1}+{a}_{-1}^{\dagger }{a}_{-1}))\mathrm{(1}-3\,{\cos }^{2}\,{\theta }_{e})\\  &  & -\frac{3}{4}({S}_{+}^{2}\,{\sin }^{2}\,{\theta }_{e}{e}^{-2i{\phi }_{e}}+H.c\mathrm{.)}\,+\,\frac{3}{2}({a}_{-1}^{\dagger }{a}_{1}\,{\sin }^{2}\,{\theta }_{e}{e}^{-2i{\phi }_{e}}+H.c\mathrm{.)}\\  &  & -\frac{3}{2}({S}_{+}{S}_{z}\,\cos \,{\theta }_{e}\,\sin \,{\theta }_{e}{e}^{-i{\phi }_{e}}+H.c\mathrm{.)}\,-\,\frac{3}{2}({S}_{-}{S}_{z}\,\cos \,{\theta }_{e}\,\sin \,{\theta }_{e}{e}^{-i{\phi }_{e}}+H.c\mathrm{.)}\\  &  & +\frac{3}{\sqrt{2}}(\cos \,{\theta }_{e}\,\sin \,{\theta }_{e}{e}^{i{\phi }_{e}}{a}_{0}^{\dagger }{a}_{1}+H.c)\,-\,\frac{3}{\sqrt{2}}(\cos \,{\theta }_{e}\,\sin \,{\theta }_{e}{e}^{-i{\phi }_{e}}{a}_{0}^{\dagger }{a}_{-1}+H.c\mathrm{.)}]\end{array}$$

Here, we have used the relations *e*_±_ = *e*_*x*_ ± *ie*_*y*_, ${e}_{\pm }=\,\sin \,{\theta }_{e}{e}^{\pm i{\phi }_{e}}$, and *e*_*z*_ = cos *θ*_*e*_.

For the Gaussian mode function

17 $$\varphi ({\bf{r}})=\frac{1}{{\pi }^{\mathrm{3/4}}\sqrt{{q}_{x}{q}_{y}{q}_{z}}}\,\exp \,[-\frac{1}{2}(\frac{{x}^{2}}{{q}_{x}^{2}}+\frac{{y}^{2}}{{q}_{y}^{2}}+\frac{{y}^{2}}{{q}_{z}^{2}})],$$

the last two terms of *H*_*d*_ vanish. After introducing the two paramters

18 $${d}_{s}=\frac{{c}_{d}}{\mathrm{4|}c|}\,\int \,\frac{d{\bf{r}}d{\bf{r}}^{\prime} {|\varphi ({\bf{r}})|}^{2}{|\varphi ({\bf{r}}^{\prime} )|}^{2}\mathrm{(1}-3\,{\cos }^{2}\,{\theta }_{e})}{{|{\bf{r}}-{\bf{r}}^{\prime} |}^{3}}$$

19 $${d}_{n}=\frac{{c}_{d}}{\mathrm{4|}c|}\,\int \,\frac{d{\bf{r}}d{\bf{r}}^{\prime} {|\varphi ({\bf{r}})|}^{2}{|\varphi ({\bf{r}}^{\prime} )|}^{2}\,{\sin }^{2}\,{\theta }_{e}{e}^{-2i{\phi }_{e}}}{{|{\bf{r}}-{\bf{r}}^{\prime} |}^{3}},$$

we obtain

20 $$\begin{array}{rcl}{H}_{d} & = & |c|{d}_{s}(3{S}_{z}^{2}+2{a}_{0}^{\dagger }{a}_{0}-{{\bf{S}}}^{2}-({a}_{1}^{\dagger }{a}_{1}+{a}_{-1}^{\dagger }{a}_{-1}))\\  &  & +|c|{d}_{n}[-\mathrm{3(}{S}_{x}^{2}-{S}_{y}^{2})+\mathrm{3(}{a}_{-1}^{\dagger }{a}_{1}+{a}_{1}^{\dagger }{a}_{-1})].\end{array}$$

Then the total Hamiltonian reduces to

21 $$\begin{array}{rcl}H/|c| & = & -{\hat{S}}^{2}+{d}_{s}\mathrm{(3}{\hat{S}}_{z}^{2}-{\hat{S}}^{2}+{\hat{N}}_{0})\\  &  & -3{d}_{n}({\hat{S}}_{x}^{2}-{\hat{S}}_{y}^{2}-{\hat{a}}_{-1}^{\dagger }{\hat{a}}_{1}-{\hat{a}}_{1}^{\dagger }{\hat{a}}_{-1}\mathrm{)}.\end{array}$$

### Dynamics of spin

Hamiltonian *H* can be expanded in Fock state basis $|{N}_{1},{N}_{0},{N}_{-1}\rangle$ with *N*_*α*_ ≥ 0 and *N*_1_ + *N*_0_ + *N*_−1_ = *N*. Numerically, it is more convenient to express Fock state basis as $|m,k\rangle$ where *m* = *N*_1_ − *N*_−1_ and *k* = *N*_1_ is the mumber of atoms in *m*_*F*_ = 1 component. Since *N*_−1_ = *k* − *m* and *N*_0_ = *N* − 2*k* + *m*, we find

22 $${\rm{\max }}\mathrm{(0},m)\le k\le [\frac{N+m}{2}].$$

Then the matrix elements of Hamiltonian *H* become ${H}_{mk,m^{\prime} k^{\prime} }\equiv \langle m,k|H|m^{\prime} ,k^{\prime} \rangle$, and the dimension is *D* × *D* with *D* = (*N* + 1)(*N* + 2)/2. The index *r* of state $|m,k\rangle$ i.e., *r*(*m*, *k*) is stored in a 1D array as

23 $$\begin{array}{ccccccc}r: & \mathrm{0,} & \mathrm{1,} & \mathrm{2,} & \mathrm{3,} & \ldots , & D-1\\ |m,k\rangle : & |-N,0\rangle , & |-N+1,0\rangle , & |-N+2,0\rangle , & |-N+2,1\rangle , & \ldots , & |N\mathrm{,\; 0}\rangle \end{array}.$$

After diagonalizing *H*, we obtain the eigenstates as |*ψ*_*s*_〉

24 $$H|{\psi }_{s}\rangle ={E}_{s}|{\psi }_{s}\rangle ,$$

if we define $|{\varphi }_{r}\rangle \equiv |m,k\rangle$ with *r* = *r*(*m*, *k*), we have $|{\psi }_{s}\rangle ={\sum }_{r}\,{u}_{r,s}|{\varphi }_{r}\rangle$ with ${u}_{r,s}=\langle {\varphi }_{r}||{\psi }_{s}\rangle$.

Assuming that the initial state takes the form $|{\rm{\Psi }}\mathrm{(0)}\rangle ={\sum }_{r}\,{f}_{r}|{\varphi }_{r}\rangle$ which is a superposition of number states, it can be expanded in the $\{|{\psi }_{s}\rangle \}$ basis as $|{\rm{\Psi }}\mathrm{(0)}\rangle ={\sum }_{s}\,{g}_{s}|{\psi }_{s}\rangle$, the time evolution of this state is

25 $$|{\rm{\Psi }}(t)\rangle =\sum _{s}\,{g}_{s}{e}^{-i{E}_{s}t}|{\psi }_{s}\rangle =\sum _{r}\,[\sum _{s}\,{u}_{r,s}{g}_{s}{e}^{-i{E}_{s}t}]\,|{\varphi }_{r}\rangle .$$

When the initial state is actually a number state $|{\rm{\Psi }}\mathrm{(0)}\rangle =|{\varphi }_{{r}_{0}}\rangle$, i.e., ${f}_{r^{\prime} }={\delta }_{r^{\prime} ,{r}_{0}}$, therefore

26 $$|{\rm{\Psi }}(t)\rangle =\sum _{r}\,[\sum _{s}\,{g}_{s,{r}_{0}}{g}_{s}{e}^{-i{E}_{s}t}]\,|{\varphi }_{r}\rangle \equiv \sum _{m,k}\,{\bar{g}}_{mk}(t)|m,k\rangle .$$
